# Evaluating the Use of UV Absorbance for the Differentiation of Humified From Non-Humified Materials

**DOI:** 10.1093/jaoacint/qsae039

**Published:** 2024-05-02

**Authors:** Mohammad Rahbari, Jarrod Psutka, Richard Lamar, Fernando L Rosario-Ortiz

**Affiliations:** BioLiNE Corporation, 3971 Old Walnut Rd., P.O Box 429, Alvinston, Ontario, N0N-1A0, Canada; BioLiNE Corporation, 3971 Old Walnut Rd., P.O Box 429, Alvinston, Ontario, N0N-1A0, Canada; Huma, Inc. 1331 W. Houston Ave., Gilbert, Arizona, 85233, USA; Department of Civil, Environmental and Architectural Engineering, University of Colorado Boulder, UCB 607, SEEC S295B, Boulder, Colorado, 80309, USA; Environmental Engineering Program, University of Colorado Boulder, UCB 607, SEEC S295B, Boulder, Colorado, 80309, USA

## Abstract

**Background:**

Products containing humic acids (HA) and fulvic acids (FA) have significant commercial potential; however, unknown to the consumer, some products may be mislabeled or contain adulterants. The prevalence of mislabeling and adulterants is found primarily in FA products. Using UV-Vis spectroscopy to differentiate between real and fake FA products is practical and desirable.

**Objective:**

The objective of this study was to expand the dataset generated using a UV-Vis-based method proposed by Mayhew et al., 2023.

**Methods:**

In total, 30 test samples were used to generate 90 test portions (three replicates per test sample) for analysis using the UV-Vis methodology outlined in Mayhew et al., 2023, which in this study is referred to as the UVAC (UV absorbance confirmation) method.

**Results:**

None of the 13 FA test samples investigated were determined as humified using the UVAC method. The FA samples studied consisted of two IHSS standards, five commercial FA products (CFAP), and six full FA fractions (SFA), which were isolated from six known solid humic material sources (SHMS). There was a leonardite, a humalite, and four peat sources used as the SHMS. Analysis of the neutralized extract of the SHMS found only 3/6 SHMS were determined as humified. Six HA (SHA) test samples were also generated by isolating the HA from the SHMS, and only 3/6 SHA were determined as humified.

**Conclusions:**

Given the high prevalence of false determinations, more work is needed to improve the method so that it can be used by industry or regulators.

**Highlights:**

The proposed method failed to determine IHSS FA standards as humified. Although the method is practical, it needs improvement and further study before it can be used for reliable differentiation of real from fake FA.

Humic substances (HS) are ubiquitous on our planet. They are naturally found in aqueous environments, soils, composts, peats, leonardite deposits (formed by oxidization of lignite coal), lignite coals, and oxidized sub-bituminous coals. They are produced in soils, composts, and wetlands through a primarily microbiological decomposition and secondary synthesis process called humification (1).

Humic substances consist of complex and heterogeneous mixtures of numerous randomly formed polydisperse macromolecular structures (1). Humic acids (HA) and fulvic acids (FA) are two operationally defined subfractions of HS that have enormous commercial potential across numerous industries, including agriculture, animal and human health, oil and gas drilling, and foundries. Humic substances have been studied extensively for their role as biostimulants in agriculture (2–6) and are becoming increasingly popular ingredients for use in pharmaceutical and human health applications, particularly FA (7–9).

There are many plant extracts, hydrolysates, and effluents from food processing, pulp and paper production, digesters, wastewater treatment, and biomass pyrolysis activities that are loaded with a heterogeneous mixture of organic molecules (e.g., poly-phenols, polysaccharides, amino acids, organic acids, lignin). Many of these materials have some physical and chemical properties similar to HA and FA, such as color, pH, and elemental analysis (10–14). These materials are sometimes intentionally mislabeled as FA or HA or used in products as adulterants. The distinction between humic substances and lignin-based products or extracts from plant residues derived from non-humified sources presents a considerable challenge for multiple industries, including agriculture and dietary supplements. This misrepresentation is enabled by the fact that, currently, there are no easy or practical analytical testing methods for identification of adulterants or non-humified materials. The inability of the current tools and methods to identify non-humified products has created an opportunity that can be exploited.

The complexity of accurately detecting these adulterants is exacerbated by the high costs associated with comprehensive testing methodologies, such as nuclear magnetic resonance (NMR). Consequently, there is an urgent need for a reliable screening method to distinguish products derived from authentic humic substances before quantification. The need presented by industry trade associations, such as the Humic Products Trade Association (HPTA), is to develop a method that screens for non-humified materials.

The prevalence of mislabeling and the use of adulterants in commercial products is found primarily in relation to FA products. Although some of these non-humified source materials may exhibit crop-beneficial properties, it is misleading and inappropriate to identify the organic constituents of these non-humic-based materials as HA or FA. Many analytical techniques with a focus on spectroscopic properties (e.g., UV-Vis absorbance, FTIR, C-NMR, EEM fluorescence) have been employed to study and differentiate humified materials with varying conclusions (15–21).

In a recently published article, Mayhew and coauthors (2023) describe a UV-spectroscopic method (UVAC) that could be used to differentiate HS from other, not humified materials (22). Key elements of the UVAC method procedures include using NaOH to create an alkaline extract from a test solid or solution followed by neutralizing (that is, adjusting the pH to 7) the alkaline extract from the previous step (22). This neutralized extract solution is then used to determine the ash-free organic matter and to obtain UV-absorbance spectra (22). UV-absorbance data are recorded every 5 nm for wavelengths from 290 nm to 330 nm inclusively (22). The analysis of the absorbance data obtained through the method includes zeroing the absorbance data by subtracting the minimum absorbance in that series from all values followed by normalizing the data by dividing by the zeroed absorbance of that sample at 290 nm (22). The scaled absorbance difference (SAD) can now be obtained by calculating the difference between the scaled absorbance value of any given aliquot analyzed with the scaled absorbance value provided for the humic standard curve that was generated by the UVAC method (22). For the SAD and the square of the sum of the scaled absorbance difference (SSSAD) calculations, the UVAC method provides users with a Humic Dashboard Spreadsheet (22). The SSSAD is calculated by summing up the differences between the sample scaled absorbance and the standard curve scaled absorbance at every 5 nm interval from 290 to 330 nm and squaring the sum. We also have included a calculation for the sum of the scaled absorbance differences (SSAD) from which the SSSAD is derived.

In the Humic Dashboard Spreadsheet, values are provided for the scaled absorbance of a standard curve. The humic standard curve was created by Mayhew et al., (2023), by taking the average of all absorbance data of neutralized alkaline extracts from five humic ores and then using the same steps to zero and obtaining the scaled absorbance values mentioned above (22). The goal, as stated by the UVAC method authors, is to normalize UV-Vis spectra internally, thus eliminating the effects of dilution and the presence of non-chromophore organic matter (22). In this study, the standard curve values provided in the Humic Dashboard were used in accordance with the procedures outlined in the method (Step 16). The SAD for each replicate aliquot was calculated using the scaled absorbance values provided in the Humic Dashboard Spreadsheet.

Using the UVAC method, Mayhew et al. (2023) reported on some promising findings for differentiating between humified and non-humified test samples. However, the study did not report on the use of the method on any IHSS standards, real commercial FA products, or the FA fraction that could be isolated from these humic ores using the classical alkaline extraction followed by acid precipitation of HA.

The International Humic Substances Society (IHSS) has developed a collection of standard HA and FA available to scientists who study HS (23). Since the early 1980s, these humic substance standards have made it possible for researchers throughout the world to compare critically their experimental results on humic substances (23). Over the decades, these IHSS standards have been used by hundreds of researchers with data reported in thousands of peer-reviewed publications. It would be ideal to include one or more IHSS FA and HA standards in the confirmation of any analytical method used for studying HS.

The purpose of the UVAC method is to determine if a test material is a HS and to reliably differentiate it from a non-HS. The UVAC method paper states that the most common misrepresentation found for HS in the marketplace is associated with FA products (22). Indeed, all three commercial FA products reported in the publication, using the UVAC method, were determined to be fake and originating from non-humic material. The authors of the UVAC method did not present any data that explicitly confirmed that the UVAC method can accurately determine an FA test sample as originating from humified or HS.

The fact that FAs are humified, and that FA is a component of HS, is widely accepted by academics, industry, and regulators (1, 3, 5–7, 9–19, 21, 22). HA and FA are operationally defined based on their behavior in acidic solutions (1). HA is the acid insoluble fraction that precipitates when the alkaline extract containing FA and HA is adjusted to pH values between 2 and 1. The organic portion that remains in solution in the acidified solutions is called FA (1). The process of neutralizing alkaline extracts of humified sources results in mixtures of HA and FA, with HA predominating. The ISO 19822 method of quantification uses an acidified hydrophobic ion-exchange resin for preferential adsorption of the hydrophobic FA fraction (24). IHSS takes this isolation one step further with a series of desalting steps, removing most inorganic ions in the material. Most products in the marketplace consist of the full fulvic fraction, which includes both the hydrophobic and hydrophilic fractions. The HA content of oxidized lignite, leonardite, and other coal ores is significantly higher than the FA content. Lamar et al. (2014) reported the average HA and FA concentration of IHSS Gascoyne Leonardite as 75.2 and 7.6%, respectively, or a 10× greater concentration of HA (25). Lamar et al. (2014) studied four other test materials referred to as D1, D2, D3, and D4 sourced from a number of mining operations in North America (25). The HA: FA ratio reported for these samples ranged from 5.5:1 to 40:1. When the alkaline extract of these ores is used to obtain UV absorbance, most of the UV absorbance is associated with the HA fractions due to their high relative concentrations compared to FA fractions (25).

Humic acid molecules have higher carbon content and are more aromatic (16,17,21,26–30) than FA molecules, which have higher oxygen content and higher concentrations of carboxylic and phenolic hydroxyl groups (17, 21, 29, 30, 31). Therefore, because the spectra of these complex mixtures are produced from the overlapping spectra of the multitude of molecular structures present and, possibly, interactions among them, the neutralized alkaline extracts used to produce the comparative spectra may not accurately represent FA spectra (20).

The main objective of this study was to expand the dataset generated using the UVAC method to include FA test samples. This study examined IHSS FA and HA standards, along with five commercial FA products, six humic ores, and four non-humic materials. An additional 12 test samples were generated by using the classical acid precipitation protocols to isolate both the HA and FA fractions of each of the six humic ores generating HA and FA test samples.

## Experimental

### Materials and Methods

The test samples that were investigated may be divided into the following five categories:

1. *IHSS standards*.—Three IHSS standards, including Suwannee River FA Standard III (SRFA), Elliott Soil FA Standard V (ESFA), and Elliott Soil HA Standard V (ESHA). There were small, inconsequential modifications made to the UVAC method for the characterization of the IHSS standards (the modifications are described in the following subsection titled “Preparing the UV-Vis Test Solutions From the IHSS Standards”).
2. *Commercial FA products (CFAP)*.—Test samples were generated from five CFAPs and measured for their UV absorbance according to the UVAC method, with no deviations in the methodology.
3. *Solid humic material sources (SHMS)*.—Test samples were generated from six SHMSs, including North Dakota Leonardite (NDL), Alberta Humalite (ABH), Canadian Sphagnum Peat (CSP), Irish Moss Peat (IMP), Balkan Black Peat (BBP), and IHSS Pahokee Peat (IPP). Humalite deposits consist of weathered subbituminous coals and carbonaceous shales that are found in Alberta. Test samples for all six SHMS source materials were generated and measured for their UV absorbance according to the UVAC method, with no deviations in the methodology.
4. *Surrogate FA (SFA) and surrogate HA (SHA) test samples*.—Some may question whether all of the CFAP (commercial FA products) tested are fake. To address this issue and to create FA test samples that are unequivocally sourced from humified sources, FA fractions were isolated from each of the six SHMSs listed above. Test samples were generated from each of the six SFA and SHA isolates and measured for their UV absorbance according to the UVAC method, with no deviations in the methodology.
5. *Non-humified materials (NHM)*.—Test samples were generated from four NHMs, including two seaweed extracts (SWE), one yucca extract (YUE), and one organic sugar cane molasses (SCM), and were measured for their UV absorbance according to the UVAC method, with no deviations in the methodology.

### Experimental Procedures for the UVAC Method

For clarity, the experimental procedures used in the UVAC method as outlined on pages 4-5 in the Mayhew et al., (2023) publication and consist of 16 steps (22). All of those 16 steps that make-up the method were followed exactly as outlined in the publication for CFAP, SHMS SFA, SHA and NHM test portions generated in this study. Small deviation to the method was used for the IHSS standards as outlined below.

### Preparing the UV-Vis Test Solutions from the IHSS Standards

Due to the high cost of the IHSS standards, test samples were prepared using smaller quantities of the IHSS standards to produce the solutions from which the 1.00 g test portion were obtained in Step 3 of the UVAC method. In Steps 8 to 10, the UVAC method requires the quantification of organic matter in the neutralized extract solution. IHSS SRFA and ESFA standards are purified hydrophobic FA fractions that are very low in ash content resulting from the isolation process used by IHSS, which includes a series of desalting steps to remove inorganics (23). Given the nature of the isolation process, there is no need to determine the organic matter content. In Step 11 of the UVAC method, the organic matter concentration measured and calculated in Step 10 is used to achieve a final concentration of 30 mg/kg of dissolved organic matter in a UV-Vis test solution for UV-Vis scanning (22).

In this study, 30 mg of IHSS standards was mixed for 30 min under N_2_ with 5.0 mL of 0.1 M NaOH solution generating a 6000 mg/kg OM solution. The pH of the solution was adjusted to 6.9, first using 3 M HCl followed by 0.6 M HCl close to the end point. This 6000 mg/kg solution was treated as the test sample made from IHSS standards for the UVAC procedures.

Test portions of this test sample generated for each IHSS standard were then subjected to Step 3 of the UVAC method in triplicate. Step 3 of the UVAC method consists of weighing out a 1.00 ± 0.05 g test portion of the test sample and adding 200 mL of 0.05 M NaOH solution and stirring constantly at 300–400 rpm for 1 h under N_2_.

For the IHSS standards, three 1 g samples were taken from the 6000 mg/kg solution prepared from the SRFA, ESFA, and ESHA in accordance with Step 3 of the UVAC method. It is important to stress that the test portions generated in Step 3 were neutralized using the exact procedures outlined in Step 5 of the UVAC method without deviation. Since we know the 1.00 g test portion used for the IHSS standards has 6000 mg/kg of ash-free organic matter, dilution was used to achieve the desired 30 mg/kg for Step 11 of the UVAC method. All subsequent steps for scanning the resulting aliquot and calculations were done with no deviation from the steps outlined in the UVAC method.

### Preparing the UV-Vis Test Solutions From the Commercial FA Products (CFAPs)

Test portions were generated for each CFAP in accordance with Step 2 of the UVAC method. All steps in the UVAC method’s experimental procedures from Steps 1 to 16 were followed with no deviation.

### Preparing the UV-Vis Test Solutions From the Solid Humic Material Sources (SHMS)

Test portions were generated for each SHMS in accordance with Step 2 of the UVAC method. All steps in the UVAC method’s experimental procedures from Steps 1 to 16 were followed with no deviation.

### Preparing the UV-Vis Test Solutions From the SFA and SHA

To prepare the SFA and SHA samples for each of the SHMS materials, 5 g of each SHMS was stirred in 0.1 M NaOH to a final volume of 1 L under a nitrogen environment for 16 h. The resulting solutions were centrifuged at 3900 *g* for 30 min, and insoluble material was discarded; 3 M HCl was used to adjust the solution to a pH of 1.0, first using 6 M HCl and then 0.6 M HCl when close to the pH target, and left undisturbed for 4 h. The solutions were centrifuged at 3900 *g* for 30 min. The soluble portion was retained as the SFA, and the insoluble portion was retained and dried at 65°C as the SHA. For each SFA and SHA test sample generated, 1 g test portions were obtained in triplicate and subjected to the UVAC method in accordance with the procedures outlined in the paper with no deviation. Table 1 provides a complete list of the test portion IDs for the test materials used in this study.

**Table 1.** Test portion IDs for materials used in the study

Category	Test portion ID	Material source and description
QCM 1.0	QCM	HPTA Quality Control Material 1.0
IHSS Standards	SRFA	IHSS, Suwannee River FA Standard III
	ESFA	IHSS, Elliott Soil FA Standard V
	ESHA	IHSS, Elliott Soil HA Standard V
Commercial FA Products (CFAP)	CFAP-1	FA Product #1
	CFAP-2	FA Product #2
	CFAP-3	FA Product #3
	CFAP-4	FA Product #4
	CFAP-5	FA Product #5
Solid Humic Material Sources (SHMS)	NDL	N.D Leonardite
	ABH	Alberta Humalite
	CSP	Canadian Sphagnum Peat
	IMP	Irish Moss Peat
	BBP	Balkan Black Peat
	IPP	IHSS Pahokee Peat
Surrogate HA Solutions (SHA)	NDL-SHA	HA isolated from ND Leonardite
	ABH-SHA	HA isolated from Alberta Humalite
	CSP-SHA	HA isolated from Cnd. Sphagnum Peat
	IMP-SHA	HA isolated from Irish Moss Peat
	BBP-SHA	HA isolated from Balkan Black Peat
	IPP-SHA	HA isolated from IHSS Pahokee Peat
Surrogate FA Solutions (SFA)	NDL-SFA	FA isolated from N.D Leonardite
	ABH-SFA	FA isolated from Alberta Humalite
	CSP-SFA	FA isolated from Cnd. Sphagnum Peat
	IMP-SFA	FA isolated from Irish Moss Peat
	BBP-SFA	FA isolated from Balkan Black Peat
	IPP-SFA	FA isolated from IHSS Pahokee Peat
Non-Humified Materials (NHM)	SWE-1	Seaweed extract #1
	SWE-2	Seaweed extract #2
	YUE	Yucca extract
	SCM	Organic sugar cane molasses

The following equipment were used: Milwaukee Mi 180 pH meter with a range of 0–14 and resolution of 0.01, Denver Instrument Company TR-204 analytical balance with readability to 0.0001 g, Quincy Lab Model 30 oven ±2°C, Pyradia F200HP muffle furnace, and Agilent 8453 UV-Vis spectroscopy system (190 to 1100 nm range, 1.0 nm spectral bandwidth/nominal spectral slit width).

Quality control material (QCM 1.0) was obtained from Mayhew, and the instructions outlined in Steps 13 and 14 of the UVAC method were followed.

### Calculation of the Square of Sum of the Scaled Absorbance Difference (SSSAD)

The Humic Dashboard Spreadsheet, provided by Mayhew et al., (2023) included data on the scaled absorbance of the humic standard curve (22). Below is a step-by-step description of the calculation procedures used to determine SSSAD in this study. The calculations follow the identical procedures outlined by the UVAC method. The first step was to obtain the UV absorbance in 5 nm increments from 290 nm to 330 nm for each aliquot, resulting in triplicate UV absorbances for each test portion listed in Table 1. For each replicate, the absorbances at each wavelength were zeroed by subtracting the value at each wavelength from the minimum absorbance in that series (22). The absorbance was scaled by dividing the zeroed absorbance at all wavelengths by the zeroed absorbance at 290 nm for that replicate. The scaled absorbance difference (SAD) was obtained by calculating the difference between the scaled absorbance value of each replicate analyzed with the scaled absorbance value provided for the humic standard curve developed by the UVAC method. The SAD values for each replicate are then summed, and the square of the sum (SSSAD) is obtained for each replicate. The final step is to average the SSSAD calculated across the three replicates done for each test portion, and if the SSSAD is >0.01, the UVAC method would conclude that this material is from non-humified source materials. Table 2 provides a sample calculation of the SSSAD for SRFA (Suwannee River FA III).

**Table 2.** Example of calculations for SSSAD based on absorbances measured for SRFA (Suwannee River FA Standard III)

Wavelength, nm	UV Abs. Rep. 1	UV Abs. Rep. 2	UV Abs. Rep. 3	Zeroed Abs. Rep. 1	Zeroed Abs. Rep. 2	Zeroed Abs. Rep. 3	Scaled Abs. Rep. 1	Scaled Abs. Rep. 2	Scaled Abs. Rep. 3	Scaled Abs. Stnd. Curve	SAD Rep. 1	SAD Rep. 2	SAD Rep. 3
290	0.360	0.286	0.297	0.154	0.127	0.127	1.000	1.000	1.000	1.000	**0.000**	**0.000**	**0.000**
295	0.337	0.267	0.277	0.130	0.108	0.107	0.847	0.854	0.841	0.868	**−0.021**	**−0.014**	**−0.027**
300	0.315	0.249	0.263	0.108	0.090	0.093	0.703	0.713	0.734	0.738	**−0.035**	**−0.025**	**−0.004**
305	0.293	0.232	0.245	0.086	0.073	0.075	0.561	0.575	0.592	0.611	**−0.050**	**−0.037**	**−0.019**
310	0.273	0.214	0.222	0.066	0.055	0.052	0.428	0.436	0.408	0.484	**−0.056**	**−0.048**	**−0.076**
315	0.254	0.198	0.201	0.047	0.039	0.031	0.305	0.311	0.243	0.360	**−0.055**	**−0.049**	**−0.116**
320	0.236	0.184	0.198	0.030	0.025	0.027	0.193	0.195	0.216	0.238	**−0.044**	**−0.042**	**−0.021**
325	0.221	0.171	0.185	0.014	0.012	0.015	0.094	0.091	0.120	0.117	**−0.024**	**−0.026**	**0.003**
330	0.207	0.159	0.170	0.000	0.000	0.000	0.000	0.000	0.000	0.000	**0.000**	**0.000**	**0.000**

										SUM:	−0.286	**−0.241**	**−0.262**
										SSSAD:	0.082	**0.058**	**0.069**
								Average SSSAD for Three Replicates:					**0.069**
								Pass/Fail (SSSAD >0.01):					**FAIL**

### Calculation and Use of Average Scaled Absorbance Difference (SAD)

In this study, ±0.1 SAD was investigated as a potential cutoff for differentiating if a test sample originated from humified or non-humified materials. The average of absorbance values measured at each wavelength was calculated for the triplicate measurements. The average absorbance was zeroed by subtracting the average measurement value at each wavelength from the minimum average in that series. The absorbances were scaled by dividing the zeroed average absorbances at each wavelength by the zeroed average absorbance at 290 nm. The scaled absorbance difference (SAD) was obtained by calculating the difference between the scaled absorbance for the test portion from the scaled absorbances for the humic standard curve. If SAD values at any given wavelength are greater than ±0.1, the test sample is determined as non-humified. Table 3 provides an example of the calculation involved for the SRFA (Suwannee River FA III).

**Table 3.** Example of calculations for SAD for use as cutoff based on absorbances measured for SRFA (Suwannee River FA Standard III)

Wavelength, nm	UV Abs. Rep. 1	UV Abs. Rep. 2	UV Abs. Rep. 3	Avg. UV Abs.	Zeroed Avg. Abs.	Scaled Avg. Abs.	Scaled Abs. Stnd. Curve	SAD
290	0.360	0.286	0.297	0.314	0.136	1.000	1.000	**0.000**
295	0.337	0.267	0.277	0.294	0.115	0.847	0.868	**−0.021**
300	0.315	0.249	0.263	0.276	0.097	0.716	0.738	**−0.022**
305	0.293	0.232	0.245	0.257	0.078	0.575	0.611	**−0.036**
310	0.273	0.214	0.222	0.236	0.058	0.424	0.484	**−0.060**
315	0.254	0.198	0.201	0.218	0.039	0.287	0.360	**−0.072**
320	0.236	0.184	0.198	0.206	0.027	0.201	0.238	**−0.037**
325	0.221	0.171	0.185	0.192	0.014	0.101	0.117	**−0.016**
330	0.207	0.159	0.170	0.179	0.000	0.000	0.000	**0.000**
				**Pass/Fail (SAD > ± 0.1):**				**PASS**

## Results and Discussion

The origin of the UV absorbance spectra of HS is an active area of research, where different theories are being evaluated regarding how the nondescriptive portion of the spectrum is obtained. One hypothesis is that multitudes of differing chemical entities are contributing to the overall observed UV absorbance, suggesting that the shape of the spectra can be obtained from a superposition of the individual spectra of the absorbing components, as demonstrated by recent work of Leresche et al. (20). There is also the expectation that some level of the overall absorbance spectra for HS is derived from interacting species (20). UV absorbance spectra for all test portions analyzed displayed similar, although not identical, featureless declining curves that are emblematic of the UV absorbance of natural organic matter (NOM).

The UVAC method falsely determined SRFA as non-humified material (Table 2). The IHSS SRFA is widely used by academic researchers and industry to investigate chemicals and structural properties of FA. Interestingly, when the SAD cutoff approach was used (Table 3), the SRFA was determined as humified material. The two calculation approaches use the exact same UV absorbance data, and yet the results contradict each other. The SAD is an intermediate step in the calculation of SSSAD. By summing the SADs across the 295–325 nm range, those samples that have small differences across multiple wavelengths in their scaled absorbance from the humic standard curve would be easier to differentiate; thus, the number of overall samples that fails increases when you sum the SAD versus just using the SAD. HA and FA have different chemical structures. It is the difference in the structural properties of these fractions of HS that results in differences in the UV absorbance of FA versus HA. These differences make it difficult to use a standard curve developed based on the neutralized extract of an ore as the comparison for the measurement and calculation of SAD for FA. For FA samples such as SRFA, the SADs remain higher across multiple wavelengths within the range. For the SRFA SAD, the absolute values for SAD at 305 to 320 nm are elevated, ranging between 0.036 and 0.072. Although these values do not exceed the ±0.1 cutoff, the sum of these SAD values is 0.264, which is much greater than the 0.1 cutoff. This discrepancy was not confined to the SRFA. For multiple FA samples studied, the SSSAD calculations resulted in a non-humic substance determination where the SAD cutoff resulted in a pass or confirmation of humic substance determination. Calculation of the SSSAD does help to increase probability of NHMs failing.

### SSSAD Calculation for IHSS Standards and Commercial FA Products (CFAP)

Table 4 provides the absorbance data for each replicate and the average of the three replicates for all test portions generated from IHSS Standards and CFAPs (commercial fulvic acid products) across the wavelengths used in the UVAC method. These absorbance values were used to calculate the SSSAD. Table 5 provides the sum of the scaled absorbance difference SSAD and the SSSAD calculated for each replicate as well as the average across the three replicates for the IHSS and CFAP test portions. The Pass/Fail column provides determination based on the >0.01 SSSAD cutoff and whether that determination matched the expected result. In this study, it is assumed that all the CFAPs contain FA from humified sources based on the claims of the manufacturer. As Table 5 indicates, the two IHSS FA standards, SRFA and ESFA, both had SSSAD values >0.01 and thus were deemed to be non-humified. The ESHA SSSAD was <0.01 and deemed to be humified by the UVAC method. All five commercial products tested had SSSAD >0.01 and deemed non-humified by the UVAC method. It should be noted that between the three commercial fulvic products tested by Mayhew et al. (2023) and the five tested in this study, not one commercial FA product was deemed humified. These results indicate false-negative determination as it relates to FA products and introduces some uncertainty for the use of the UVAC method on commercial FA products. The fact that both IHSS FA standards were deemed to be non-humified is problematic for the use of the UVAC method by industry and regulators. To develop a better understanding of whether the UVAC method can determine any FA solution as humified, surrogate FA (SFA) products were generated by isolating the full FA fraction from the six SHMS. The classical acid precipitation method was used to separate the HA and FA fractions.

**Table 4.** UV Absorbance for each replicate for IHSS standards and CFAPs

Category	Test portion ID		290 nm	295 nm	300 nm	305 nm	310 nm	315 nm	320 nm	325 nm	330 nm
IHSS Standards	SRFA	Rep1	0.360	0.337	0.315	0.293	0.273	0.254	0.236	0.221	0.207
	SRFA	Rep2	0.286	0.267	0.249	0.232	0.214	0.198	0.184	0.171	0.159
	SRFA	Rep3	0.297	0.277	0.263	0.245	0.222	0.201	0.198	0.185	0.170
	SRFA	Avg.	0.314	0.294	0.276	0.257	0.236	0.218	0.206	0.192	0.179
	ESFA	Rep1	0.351	0.328	0.306	0.286	0.266	0.247	0.229	0.211	0.196
	ESFA	Rep2	0.338	0.310	0.285	0.262	0.241	0.221	0.208	0.195	0.177
	ESFA	Rep3	0.337	0.308	0.286	0.263	0.252	0.231	0.210	0.197	0.183
	ESFA	Avg.	0.342	0.315	0.292	0.271	0.253	0.233	0.216	0.201	0.185
	ESHA	Rep1	0.377	0.364	0.352	0.339	0.326	0.312	0.299	0.286	0.275
	ESHA	Rep2	0.395	0.382	0.369	0.356	0.342	0.327	0.313	0.298	0.285
	ESHA	Rep3	0.378	0.365	0.352	0.340	0.326	0.313	0.299	0.288	0.277
	ESHA	Avg.	0.383	0.370	0.358	0.345	0.331	0.318	0.304	0.291	0.279
Commercial FA Products (CFAP)	CFAP-1	Rep1	0.126	0.116	0.107	0.098	0.089	0.081	0.074	0.067	0.061
	CFAP-1	Rep2	0.126	0.114	0.108	0.100	0.090	0.083	0.076	0.069	0.062
	CFAP-1	Rep3	0.126	0.115	0.107	0.100	0.090	0.082	0.075	0.069	0.062
	CFAP-1	Avg.	0.126	0.115	0.107	0.099	0.090	0.082	0.075	0.068	0.062
	CFAP-2	Rep1	0.235	0.196	0.174	0.159	0.147	0.137	0.129	0.122	0.115
	CFAP-2	Rep2	0.220	0.184	0.171	0.150	0.139	0.130	0.121	0.115	0.110
	CFAP-2	Rep3	0.240	0.197	0.175	0.158	0.147	0.138	0.129	0.122	0.115
	CFAP-2	Avg.	0.232	0.192	0.173	0.156	0.144	0.135	0.126	0.120	0.113
	CFAP-3	Rep1	0.744	0.676	0.619	0.566	0.518	0.473	0.434	0.398	0.364
	CFAP-3	Rep2	0.750	0.689	0.620	0.573	0.528	0.478	0.440	0.398	0.365
	CFAP-3	Rep3	0.766	0.691	0.631	0.579	0.532	0.483	0.448	0.401	0.368
	CFAP-3	Avg.	0.754	0.686	0.624	0.573	0.526	0.478	0.441	0.399	0.366
	CFAP-4	Rep1	0.562	0.518	0.475	0.434	0.395	0.358	0.324	0.293	0.264
	CFAP-4	Rep2	0.585	0.539	0.493	0.450	0.409	0.369	0.334	0.301	0.271
	CFAP-4	Rep3	0.584	0.538	0.492	0.449	0.408	0.369	0.333	0.301	0.271
	CFAP-4	Avg.	0.577	0.532	0.487	0.444	0.404	0.365	0.330	0.298	0.269
	CFAP-5	Rep1	0.230	0.200	0.175	0.166	0.143	0.132	0.126	0.083	0.052
	CFAP-5	Rep2	0.235	0.202	0.176	0.166	0.143	0.132	0.126	0.109	0.076
	CFAP-5	Rep3	0.232	0.200	0.177	0.162	0.146	0.133	0.126	0.090	0.062
	CFAP-5	Avg.	0.232	0.201	0.176	0.165	0.144	0.132	0.126	0.094	0.064

**Table 5.** SSSAD determination for IHSS standards and CFAPs

Category	Test portion ID		SSAD	SSSAD	Pass/Fail	Expected result
IHSS Standards	SRFA	Rep1	−0.286	0.082	Fail	No
	SRFA	Rep2	−0.241	0.058	Fail	No
	SRFA	Rep 3	−0.262	0.069	Fail	No
	SRFA	Avg.	−0.263	0.069	Fail	No
	ESFA	Rep1	−0.241	0.058	Fail	No
	ESFA	Rep2	−0.241	0.058	Fail	No
	ESFA	Rep 3	−0.245	0.060	Fail	No
	ESFA	Avg.	−0.242	0.059	Fail	No
	ESHA	Rep1	0.066	0.004	Pass	Yes
	ESHA	Rep2	0.149	0.022	Fail	No
	ESHA	Rep 3	0.006	0.000	Pass	Yes
	ESHA	Avg.	0.074	0.009	Pass	Yes
Commercial FA Products (CFAP)	CFAP−1	Rep1	−0.269	0.072	Fail	No
	CFAP−1	Rep2	−0.200	0.040	Fail	No
	CFAP−1	Rep 3	−0.238	0.057	Fail	No
	CFAP−1	Avg.	−0.236	0.056	Fail	No
	CFAP−2	Rep1	−1.230	1.513	Fail	No
	CFAP−2	Rep2	−1.227	1.506	Fail	No
	CFAP−2	Rep 3	−1.317	1.735	Fail	No
	CFAP−2	Avg.	−1.258	1.585	Fail	No
	CFAP−3	Rep1	−0.426	0.182	Fail	No
	CFAP−3	Rep2	−0.376	0.142	Fail	No
	CFAP−3	Rep 3	−0.434	0.188	Fail	No
	CFAP−3	Avg.	−0.412	0.170	Fail	No
	CFAP−4	Rep1	−0.236	0.056	Fail	No
	CFAP−4	Rep2	−0.238	0.057	Fail	No
	CFAP−4	Rep 3	−0.248	0.061	Fail	No
	CFAP−4	Avg.	−0.241	0.058	Fail	No
	CFAP−5	Rep1	0.292	0.085	Fail	No
	CFAP−5	Rep2	−0.137	0.019	Fail	No
	CFAP−5	Rep 3	0.114	0.013	Fail	No
	CFAP-5	Avg.	0.090	0.039	Fail	No

### SSSAD Calculations for Solid Humic Material Sources (SHMS)

Using the SSSAD calculated according to the UVAC method, three out of six SHMS test samples measured were determined to come from a humified source. Those that passed included: NDL (North Dakota Leonardite), CSP (Canadian Sphagnum Peat), and the IPP (IHSS Pahokee Peat). Table 6 provides the summary data for all test samples generated for solid humic material sources (SHMS), surrogate humic acids (SHA), surrogate fulvic acids (SFA), and non-humified materials (NHM) analyzed in this study. The table provides the average SSAD and SSSAD calculated for the three replicate test portions for each test sample. Using the SSSAD >0.01 cutoff, test samples that “pass” are determined to be humified, and those test samples that “fail” are determined as non-humified. For all replicate absorbance data and calculations, please *see* the Supplemental Information—Data Tables and Calculations.

**Table 6.** SSSAD for SHMS, SHA, SFA, and NHM test portions

Category	Test sample ID	Avg. SSAD	Avg. SSSAD	Pass/Fail	Expected result
Solid Humic Material Sources (SHMS)	NDL	−0.113	0.013	Pass	Yes
	ABH	−0.378	0.143	Fail	No
	CSP	0.061	0.004	Pass	Yes
	IMP	0.228	0.053	Fail	No
	BBP	0.190	0.039	Fail	No
	IPP	−0.038	0.002	Pass	Yes
Surrogate HA Solutions (SHA)	NDL-SHA	−0.090	0.008	Pass	Yes
	ABH-SHA	−0.499	0.250	Fail	No
	CSP-SHA	0.082	0.007	Pass	Yes
	IMP-SHA	−0.416	0.179	Fail	No
	BBP-SHA	−1.111	1.293	Fail	No
	IPP-SHA	−0.038	0.002	Pass	Yes
Surrogate FA Solutions (SFA)	NDL-SFA	−0.104	0.041	Fail	No
	ABH-SFA	−0.646	0.487	Fail	No
	CSP-SFA	0.435	0.190	Fail	No
	IMP-SFA	0.327	0.109	Fail	No
	BBP-SFA	0.633	0.432	Fail	No
	IPP-SFA	−0.281	0.079	Fail	No
Non Humified Materials (NHM)	SWE-1	−0.401	0.367	Fail	Yes
	SWE-2	−0.769	0.600	Fail	Yes
	YUE	−0.769	0.596	Fail	Yes
	SCM	0.447	0.200	Fail	Yes

### SSSAD Calculations for Surrogate HA (SHA)

It is not surprising that the SHA (surrogate HA) extracted from NDL, CSP, and IPP passed as expected (Table 6), whereas the HA from ABH, IMP, and BBP failed. These were the same sources of SHMS that passed. That is to say, the absorbance data for the HA fraction extracted from the six SHMS was similar to the absorbance for the neutralized extract of the ore where all soluble organic carbons including both HA and FA were present in the test solutions generated. This further supports that the HA fraction is dominating the absorbance measurements for test solutions that contain both HA and FA fractions at the neutral pH used in the UVAC method.

### SSSAD Calculations for Surrogate FA (SFA)

All six (6 out of 6) of the SFA test samples prepared failed to be determined as coming from humic sources. Combining the results of the SFA with the IHSS FA standards and the CFAP, there was a total of 13 FA test samples tested in this study, all of which failed to be determined as humified. It is noteworthy to reiterate that these SFA samples were isolated from known humic ores and peat that are commonly used sources of FA for commercial products. The method used to isolate the SFA is a well-established method of precipitating HA in acidic solution and using a centrifuge to separate the HA from the FA fractions. These surrogates are real FA and should have passed the UVAC method. The failure of the UVAC method to accurately determine FA is not entirely unexpected given the well-documented differences in the chemical structure of FA molecules versus HA molecules. There is wide acceptance among researchers as demonstrated by multiple publications that, in general, HA fractions tend to have a higher degree of aromaticity and less functionality than FA fractions of humic substance (25–32). The use of UV absorbance measurements and ratios for comparing the degree of aromaticity of different humic substances is also well established in previous research (25–32). The higher concentration of HA molecules in the alkaline extracts of humic ores results in HA fractions dominating the UV absorbance properties. Focusing on the absorbance measured in the range between 290 and 330 is an excellent approach to reduce variability in UV absorbance measured. Zeroing and scaling the absorbance is also very effective in that endeavor. Although the approach helps to minimize the difference between HA and FA absorbance curves, the SAD difference between FA and HA remains substantial enough to cause the SSSAD calculations to be above the cutoff.

### SSSAD Calculations for Non-Humic Materials (NHM)

This is the only category that resulted in all four test materials meeting the expected results. All four NHM samples failed the test and were accurately determined to be non-humified when using the SSSAD calculations.

### Using the Average SAD for the Determination

As mentioned in the “Materials and Methods” section, in this study a ±0.1 SAD was also investigated as a potential cutoff for differentiating if a test sample originated from humified or non-humified materials. The scaled absorbance difference (SAD) was obtained by calculating the difference between the scaled absorbance for the test portion by the scaled absorbances for the humic standard curve. Examples for the calculation were provided in Table 3. The results for this approach are provided in Table 7 for all test samples used in this study. As the data in Table 7 indicate, using this approach increases the success rate, although it still leads to both false-positive and false-negative determinations. Using the SSSAD approach with a >0.01 cutoff, only 7 of 26 test samples from humified materials resulted in an expected “pass” result. Using the SAD approach with a ±0.1 cutoff, the success rate was increased to 19 of 26 humified samples. Using the SAD approach, both IHSS FA test samples passed, as well as 3 of 6 surrogate FA samples (SFA) and 2 of 5 CFAP (commercial FA) products. Observing the SAD plots generated by the test samples, it was clear that for many FA products the SAD values approached the ±0.03 to 0.1 range but did not exceed it. The absolute value of the SAD remains elevated across multiple wavelength readings, resulting in a sum of the SAD values across the range of wavelengths being greater than 0.1. Therefore, by using the square of the sum of the SAD values (SSSAD) for FA samples, the results will be greater than 0.01. This summation results in more samples failing, which increases the probability that an NHM will fail the test, but it also results in FA failing the test.

**Table 7.** Using the SAD method for all test portions

Category	Test sample ID	Max SAD (+/-)	Pass/Fail	Expected result
IHSS Standards	SRFA	0.072	Pass	Yes
	ESFA	0.046	Pass	Yes
	ESHA	0.023	Pass	Yes
Commercial FA Products (CFAP)	CFAP-1	0.046	Pass	Yes
	CFAP-2	0.253	Fail	No
	CFAP-3	0.077	Pass	Yes
	CFAP-4	0.047	Fail	No
	CFAP-5	0.132	Fail	No
Solid Humic Material Sources (SHMS)	NDL	0.019	Pass	Yes
	ABH	0.075	Pass	Yes
	CSP	0.016	Pass	Yes
	IMP	0.045	Pass	Yes
	BBP	0.046	Pass	Yes
	IPP	0.022	Pass	Yes
Surrogate HA Solutions (SHA)	NDL-SHA	0.030	Pass	Yes
	ABH-SHA	0.092	Pass	Yes
	CSP-SHA	0.018	Pass	Yes
	IMP-SHA	0.090	Pass	Yes
	BBP-SHA	0.248	Fail	No
	IPP-SHA	0.018	Pass	Yes
Surrogate FA Solutions (SFA)	NDL-SFA	0.022	Pass	Yes
	ABH-SFA	0.131	Fail	No
	CSP-SFA	0.122	Fail	No
	IMP-SFA	0.071	Pass	Yes
	BBP-SFA	0.138	Fail	No
	IPP-SFA	0.054	Pass	Yes
Non Humified Materials (NHM)	SWE-1	0.087	Pass	No
	SWE-2	0.164	Fail	Yes
	YUE	0.166	Fail	Yes
	SCM	0.094	Pass	No

There was a disadvantage to using the ±0.1 SAD cutoff compared to calculating the SSSAD. For some NHMs (non-humified materials), the calculated SAD will also be elevated but smaller than the ±0.1 cutoff. This approach increases the pass determination for FA and NHM, resulting in false-positive determinations of NHMs. In this study, 2 of 4 NHMs resulted in false-positive determinations using the SAD approach, whereas there were none using the SSSAD.

As discussed previously and acknowledged in (22), the mislabeling and adulteration of FA products necessitate an analytical method that could be used by industry and regulators to confidently verify humic substances (22). It is critical that the method does not result in false-negative or false-positive determination; thus, more work is needed for the use of the UVAC method with FA products. After all, most consumers are not buying ores and doing an alkaline extract themselves. Most products labeled FA are not neutralized alkaline extracts of HS. Many manufacturers are further processing and purifying extracts to produce FA products, and, as such, any method proposed for use by industry and regulators needs to account for the detection of purified FA.

## Conclusions

In this study, a total of 30 test samples were prepared and investigated using the UVAC method proposed by Mayhew et al. (2023). Using the UVAC method to calculate the SSSAD for two IHSS FA standards (SRFA and ESFA), five commercial FA products (CFAP)s, and six surrogate FA (SFA) test samples resulted in non-humic determination for all of them. The false-negative determination of FA found in this study raises uncertainty regarding whether the UVAC method is fit for use by industry or regulators. This study found that by using a cutoff value of 0.1 for the scaled absorbance difference (SAD), both IHSS FA standards, as well as 3 of 6 SFA and 2 of 5 CFAP, for a total of 7 of 13 FA test samples, passed. Although the SAD cutoff increased the pass rate for FA, this approach also increases the pass rate for non-humified materials (NHMs), resulting in false-negative determinations, with 2 out of 4 NHMs passing when they should not have. More work is needed to improve the reliability of the UVAC method, especially when it comes to analyzing fulvic acid products.

## Supplementary Material

qsae039_Supplementary_Data
